# Reaching the Ball or Missing the Flight? Collective Dispersal in the Two-Spotted Spider Mite *Tetranychus urticae*

**DOI:** 10.1371/journal.pone.0077573

**Published:** 2013-10-15

**Authors:** Gwendoline Clotuche, Maria Navajas, Anne-Catherine Mailleux, Thierry Hance

**Affiliations:** 1 Earth and Life Institute, Biodiversity Research Centre, Université catholique de Louvain, Louvain-la-Neuve, Belgium; 2 INRA, UMR CBGP (INRA/IRD/Cirad/Montpellier SupAgro), Campus International de Baillarguet, Montferrier-sur-Lez, France; Sheffield University, United States of America

## Abstract

The two-spotted spider mite is a worldwide phytophagous pest displaying a peculiar dispersal. At high density, when plants are exhausted, individuals gather at the plant apex to form a collective silk-ball. This structure can be dispersed by wind or phoresy. Individuals initiating the ball are enclosed in the centre and have a high risk to die. For the first time, the ultimate and proximate mechanisms leading to this group dispersal are examined. To explore if a particular mite genotype was involved in the ball formation, plants were infested with individuals of different genetic background. After the silk-ball formation, the mites in the ball and those remaining on the plant were collected and genotyped. The balls were harvested after 4h and 24h to determine the role of timing between the formation and dispersal on the mortality of mites. Mites do not segregate according to their degree of relatedness, stage, or sex. Mites parallel humans using public transportation: they climb up in the ball whatever their genetic background. Silk-balls composed of unrelated individuals may help avoiding inbreeding when colonizing a new plant. Our results also emphasize the importance of an adequate timing for efficient dispersal between the time spent between ball formation and dispersal.

## Introduction

When a given environment becomes non-viable (e.g. food shortage or climatic constraints), individuals have to find better opportunities elsewhere by dispersal [1,2]. Processes of dispersal are considered a key factor in the survival of most organisms by determining their distribution, group organization, population dynamics, and genetic structure [3,4]. The mode of dispersal varies between species, stage of the life cycle, sex, external/social environments, and time [1,5]. 

Collective dispersal are common in gregarious species, including humans [6,7]. However, proximate and ultimate causes (i.e. how biological traits operate in an animal and why an animal behaves the way it does) that drive an individual to disperse and to follow the group or not are poorly understood [8,9]. Benefits of collective dispersal may be diverse depending on the situation: inbreeding avoidance, overcoming Allee effects (positive density dependence) [10,11], indirect fitness gain (kin selection theory) [12], access to mates, resource competition [1,13], or decreased predation risk [14]. On the other hand, the main costs of collective dispersal are related to ecological traps (i.e. scenarios in which rapid environmental change leads organisms to settle in poor-quality habitats), bad habitat choices [15,16] or to an increasing risk of inbreeding if collective dispersal is linked to degree of relatedness. It remains however unclear how individuals optimize trade-offs between costs and benefits of dispersal and thus how, and under what ecological conditions, different dispersal strategies evolve [8].

In this paper, we examine the proximate and ultimate mechanisms leading to a collective dispersal in the two-spotted spider mite, *Tetranychus urticae*, when food resources become scarce as a result of overcrowding conditions. This mite exhibits some levels of social life, such as collective web building [17,18] and a positive group effect [19]. Moreover, *T. urticae*, like other arthropod species [20-22] can discriminate between kin and non-kin [23-26]. 

Efficient dispersal behaviour has been described for this mite. Individuals can disperse alone either by crawling [27] or by aerial dispersal (winds) [28,29]. When plants become overcrowded and food resources become scarce, individuals can disperse collectively by the formation of silk-balls [30-32]. While individual modes of dispersal (i.e. ambulatory and aerial dispersal) are restricted to inseminated females [28], the silk-balls mainly contained immature individuals [32]. Different dispersal strategies between adults and juveniles seem therefore to occur in this species (i.e. phenotype-dependent dispersal).

The formation of silk-balls results from a recruitment process (with silk as the amplification cue), which progressively conglomerates thousands of mites [32]. This collective dispersal however, might be risky as mites initiating the ball are rapidly trapped within a dense silk network. After one-day of formation, most of the individuals in the centre of the silk-ball are dead: only the last mites reaching the ball stay alive. Some mites do not reach the ball and remain on the plant [32]. Two non-exclusive hypotheses can explain the process leading to the silk-ball formation. First, silk-balls could correspond to a form of altruism, which may be explained by kin selection. Indeed, individuals initiating the balls are situated at the centre of the structure. They are quickly surrounded by silk and finally die during the process. As *T. urticae* can recognize their relatives [23-26], cooperation and kin benefits could then come into play by favouring aggregation. In this case, genetically related mites could aggregate preferentially in the ball [25]. Second, the silk-ball could aggregate related and unrelated individuals, and could serve as a means to leave the plant when there is a shortage of food. Balls can reach a certain size (i.e. when it contains enough individuals) to be easily transported (by wind or animals) or to avoid negative Allee effect at low density during the early colonization stages. In this case, aggregation in the ball will not necessarily depend on the genetic relatedness among dispersing individuals and the silk-ball strategy would be selected by natural selection. The silk-ball structure represents their last chance of survival even if the death probability increases with the delay between the ball formation and its dispersal.

To understand the mechanisms that underlie the emergence and growth of the ball, we artificially infested plants with two lines of mites. Then, we examine their distribution and genetic relatedness in the balls, in the leaves and between the two groups. More precisely, we ask what proportion of the mites in the infested plant reaches the ball and what is the probability of dying related to the delay between ball formation and its dispersal. We examine the degree of relatedness of individuals, and also the sex of mites inside the ball and how they differ from those remaining on the infested plant. To address these questions, we used two methods to trace individuals during the formation of collective silk-balls. One is based on the genotype of the mites by using microsatellite markers. The other uses a phenotypic marker, the body colour of two different strains. The two markers (genotype or colour) allowed us to differentiate the mites and to assess their distribution in the ball and in the plant and to follow them during the process of silk-formation. 

## Materials and Methods

### Plant growth conditions

**Figure 1:**
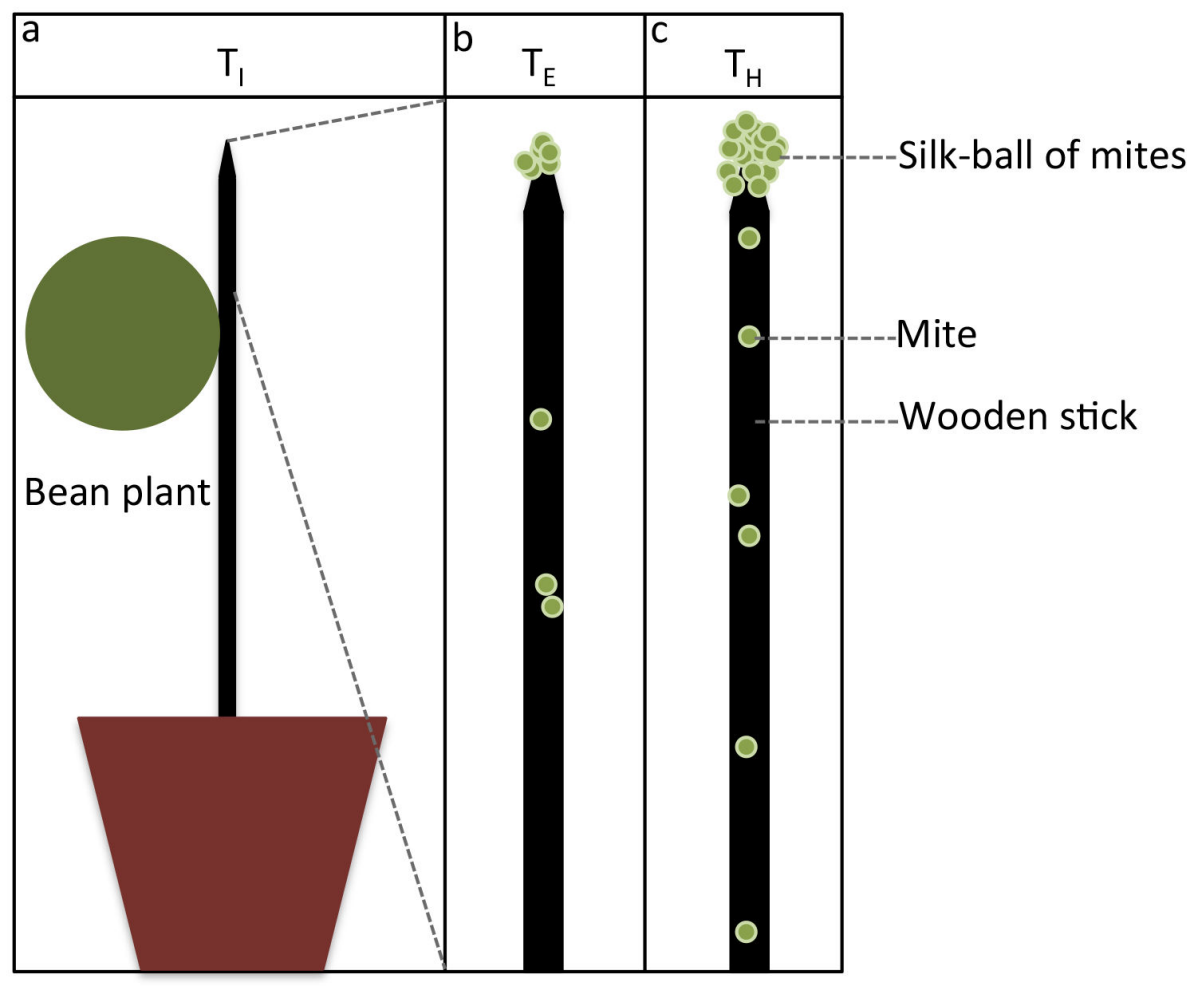
Experimental set-up. a) Plant infestation with A_0_ mites (infestation time, T_I_), b) Emergence of the silk-ball at the stick apex (emergence time, T_E_) and, c) Harvesting of the silk-ball after 24 or 4 hours (harvest time, T_H_).

Bean plants used in this experiment were reared in a growth chamber under standardized conditions (20.6 °C, 24% relative humidity R.H.). Plants of 10.5-11 cm high (2 first leaves) were used. One leaf per plant was removed. The remaining leaf was cut into a disc with a diameter of 5.5 cm (Figure 1). During the whole experiment, only this “disc leaf” was kept on the plant, all the other shoots being cut daily.

### Mites

To distinguish individuals during the ball formation, two different strains displaying distinctive colours were used: URT (green) and CAR (red). The two strains also had distinctive genetic backgrounds (*F*_*ST*_ = 0.46) [33]. The URT strain was collected in October 2005 from greenhouse-grown banana leaves (Louvain-la-Neuve, Belgium). The CAR strain was collected in January 2006 from orchard-grown citrus leaves (Tunis, Tunisia). 

However, as these two strains were coming from different geographical locations (Belgium and Tunisia), they could differ in their dispersive behaviour because of their evolutionary history. In consequence, we designed a second experiment using two isofemale lines (called thereafter as IF1 and IF2) from the LS-VL strains (i.e. offspring from single females mated to their own sons; see Information S1 for more details). The two lines were recognizable using genetic markers. The strain LS-VL (green body colour) was collected in October 2000 from a rose garden (Ghent, Belgium) [34]. Mother–son mating over two generations were repeated to increase homogenization and genetic divergence between lines. 

In the laboratory, strains and isofemale lines were reared in separated cages on bean plants (*Phaseolus vulgaris* L.), a preferred host plant of *T. urticae* [35]. Breeding stocks were maintained in a climate room at 26 °C with a relative humidity of 50–60% and a photoperiod of L16:D8. 

## Experimental Procedures

### Experiment I - Two strains: Silk-ball formation and population distribution between silk-balls and leaves

#### Phase 1, Plant infestation with mites

Bean plants were placed individually in a cage (50 × 50 × 40 cm) under standardized conditions (26 °C, 33% R.H.). A black wooden stick (25 cm high, 3 mm in diameter) was attached to the stem of each plant (Figure 1). The base of the stick was buried in the soil leaving a height of 20 cm above soil level and attached to the stem with a piece of plastic wire [32]. Thirty-five adult females (called A_0_) from each strain, either red or green, were randomly chosen from the rearing stocks and deposited on the tested plant (infestation time, T_I_, N = 13) (Figure 1a) for a period of 24 h (egg laying period). 

#### Phase 2, Silk-balls formation and harvest

After approximately 10 days, eggs matured into adults (called A_1_) and were allowed to grow on bean plants. When plants became exhausted and overcrowded (about 15 days after infestation of the bean plant), a part of the individuals gathered at the apex of the plants and started to form a collective structure embedded in silk (silk-ball emergence T_E_, Figure 1b). The ball was considered as emerged when at least 10 mites were permanently aggregated on top of the wooden stick (some of them were already embedded in silk). Before the formation of the ball, the mites that reached the apex immediately started their descent back to the plant: they did not stay at the apex. The increase of the silk deposited on the stick was therefore doubled, which stimulated the departure of new mites, creating a positive feedback (recruitment process) and ultimately the formation of the ball [32]. To collect the mites from the ball (24 h after the emergence of the ball), the wooden stick was carefully removed from the plant and the ball was gently removed from the apex with tweezers (harvest time, T_H_, Figure 1c; N = 13). 

#### Phase 3, Population characterization of silk-balls and bean leaves

To characterize the individuals forming the ball and those remaining on the plant, we separately placed the ball and the leaf on a fresh bean leaf (a disc 32 mm in diameter placed on damp cotton in a Petri dish). 

After the dead mites of the ball were counted (number, sex, stage, and colour), living (mobile) mites were allowed to mature on the bean leaf (about 10 days). Because most of the mites were immature (larvae without any distinctive body colour) [32], they needed to reach the adult stage before being sorted according to colour, either green or red. Males of both strains could not be differentiated because they had the same body colour. Mature mites were counted daily (number, sex, and colour) and removed from the bean leaf until all mites from the ball were counted. 

Bean “disc leaves” infested with mites (5.5 cm diameter, Figure 1a) were placed individually on a fresh bean leaf for 24 h. After this period of time, all mobile mites from infested “disc leaves” migrated (i.e. by ambulatory displacement) to the fresh bean leaf. The number of mites was then counted daily (number, sex, and colour).

### Experiment II - Two isofemale lines: Silk-ball formation and mites distribution between silk-balls and leaves

#### Phase 1, Plant infestation with mites

As for Experiment *I*, thirty-five adult females (A_0_) from each isofemale line (IF1, IF2) randomly chosen from the rearing stock were deposited on the tested plant (infestation time, T_I_, N = 3) (Figure 1a) for a period of 24 h. We collected the ball 4 h after its formation to observe whether the time of harvesting influences the proportion of dead mites (and not after 24 h as in Experiment *I*, Figure 1c). At the end of the formation of the ball, mature females were collected in silk-balls and kept in absolute ethanol (-20 °C) until genetic analysis (N = 3 balls). 

#### Phase 2, Silk-balls formation and harvest

The same protocol described for Experiment *I* was used. After 4 h of ball formation, balls were gently removed from the apex with tweezers (harvest time, T_H_, Figure 1c, N = 3). 

#### Phase 3, Population characterization of silk-balls and bean leaves

As described for Experiment *I*, balls and leaves were placed on fresh bean leaves and the number and sex of mites was counted daily. Because females of isofemale lines were all green, it was impossible to differentiate them using body coloration criterion.

#### Mite genotyping

Almost all individuals in both, the ball and the plant (bean leaf) of each replicate, could be genotyped (199, 191 and 125 mites, N = 3 experiments). Additionally, 30 individuals from both isofemale lines (IF1, IF2) were genotyped. DNA was extracted from single mites with the DNeasy Tissue Kit (Qiagen®) and eluted in 200 µL of AE elution buffer. Twenty microsatellite markers previously developed for *T. urticae* [35,36] were amplified in two multiplex PCR reactions (Table 1 and Information S2). Either the forward or the reverse primer for each locus was 5’ end labelled with one fluorescent dye (either FAM, NED, PET, or VIC). A total reaction volume of 10 µl, containing 0.02 µM of each primer and 4 µL of extracted DNA, was used with the QIAGEN Multiplex PCR Kit following the manufacturer's protocol. After an initial denaturation step at 95 °C for 15 min, 30 cycles were carried out, consisting of 30 s denaturation at 94 °C, 90 s at annealing temperature (52 °C or 58 °C depending on the marker set, Table 1) and 60 s extension at 72 °C, followed by a final 30 min extension step at 60 °C. PCR products were detected using an ABI PRISM 3130xl (Applied Biosystems). The PCR product was diluted in water (2 µL of the PCR product diluted in 150 µL of water). Then, 2 µL of this diluted PCR product was mixed with 19.92 µL of Hi-Di^TM^formamide (Applied Biosystems) and 0.08 µL of GeneScan^TM^ 500 LIZ® Size Standard (Applied Biosystems).

**Table 1:** Microsatellite loci assembled in two multiplex PCR sets, and used to genotype *Tetranychus urticae* mites (details on microsatellite isolation and characterization are described in [35,36], see Information S2).

**Locus**	**Core repeat**	**Size of cloned allele (bp)**	**Size range (bp)**	**Fluorescent label**	**Primer (µM**)	**Primer sequence (5'-3')**	
**PCR multiplex set 1 (Annealing temperature: 58°C)**						
*TuCA72*	(GT)_6_	262	264-270	VIC	0.02	F: ACTGTCTGGCGTCTTTTGTC	
						R: GTTTACCAGTTTCCCTTTGACC	
*TuCT37*	(TC)_7_C(TC)_5_	128	121-127	VIC	0.02	F: GTAATAATGGGTGTTGTTGC	
						R: CGCAAAATATGAGTGAGAATG	
*Tu11*	(GT)_29_	154	148-168	PET	0.02	F: CGTGTACAATCAGTCAACATCC	
						R: TGGACTTTTTAACCGTGGCT	
*Tu27*	(GA)_9_	66	57-84	PET	0.02	F: TCAAACTTGCTTTTTTCAC	
						R: GTGATGAAAATGGAGATGG	
*TuCA12*	(CA)_7_	276	263-275	FAM	0.02	F: GATTTGTGGTCGTGGTTTTC	
						R: GATCAACTCAAAAGGATAACGTTG	
*TuCT09*	(CT)_15_	129	102-128	FAM	0.02	F: GATCACTTTTTCATGTTATTCTG	
						R: CTTGGAATGAACTTTAGCAC	
*TuCA25*	(TC)_13_	164	155-165	FAM	0.02	F: AATGTGTTGGTTGTTTACGAAGTG	
						R: TTGGTCAAAGCCGGTTACAG	
*TuCT13*	(TC)_7_	164	163-168	NED	0.02	F: CAGATGGATTTGCTTTCCAC	
						R: GATCCTAATCAACATGAGGGTC	
*TuCT81*	(TC)_9_	346	345-350	NED	0.02	F: GATTCGTGAAGCCGATATTGA	
						R: GTCATCATCGTTACAAATTCTG	
*TuCT67*	(CT)_9_	95	95-107	NED	0.02	F: CCATCATCTTCATCATTCTTCACC	
						R: TAGAACAGTCAAGCAAAAAGAGTC	
**PCR multiplex set 2 (Annealing temperature: 52°C)**						
*TuCT73*	(CA)_7_	111	111-113	VIC	0.02	F: CGATGTGGGTGGTAAGCATG	
						R: ACGATGATATTGATGATGAGCG	
*TuCA83*	(GT)_6_	205	206-208	VIC	0.02	F: CAGGGTGAAACTTAGATACC	
						R: CAATTTTCCCTCTACATCTC	
*Tu16*	(GAT)_7_	136	130-148	PET	0.02	F: TTCCAATGGAAAGTGGATTTG	
						R: TCAATACCAAACGAAACTGGC	
*Tu1*	(GT)_30_	152	144-182	PET	0.02	F: GATGTAAAGGAGCGCAAAGG	
						R: CATTGTTTGTCGATTTCCTCTC	
*TuCT17*	(GA)_17_	318	288-320	NED	0.02	F: GTGATTGTTTGGCGTGCTTAG	
						R: CATTGACCAGAGCGACATTG	
*TuCT26*	(GA)_8_	154	150-156	NED	0.02	F: CGATGGAGCCGTTTCAAGAG	
						R: TCGTCATCATTGCCGTCATTTTAC	
*Tu35b*	(TGA)_8_	108	99-120	NED	0.02	F: AATGGAATGAGTTATCGTTGGG	
						R: CTTCCCGAAGGCTGTTGATA	
*TuCT18*	(CT)_9_	296	294-296	FAM	0.02	F: CTTGATGCTAGTGATACAACG	
						R: CAAGGTGATGATTTGATTTAAAG	
*TuCT04*	(CT)_8_	149	146-152	FAM	0.02	F: CGTCATCATTGCCGTCATTTTAC	
						R: GGAGCCGTTTCAAGAGAGTG	
*TuCA96*	(TG)_7_	111	107-109	FAM	0.02	F: ATGGATTGTCACCGATTTCA	
						R: CTGAAGTTTACTTGCTATAGTC	

## Data Analysis

### Distribution of mites (number, strain, sex) in the ball and on the leaf

Because data obtained during our observations were normally distributed, we used only parametric tests. For both experiments (I and II), t-tests were used to determine whether the distribution of mites (A_1_, silk-ball formation: number of mites in balls and leaf discs) was equal. For *experiment I*, t-tests were also used to determine whether green and red mites were homogeneously distributed. Tests were performed using GraphPad Prism version 5.01 for Windows (GraphPad Software, San Diego, California, USA, http://www.graphpad.com). 

### Population differentiation obtained by the isofemale lines approach (Experiment II)

Alleles were assigned using the software Genemapper 4.0 (Applied Biosystems). Pairwise *F*_*ST*_ values were calculated for each population comparison (N = 3) and for the isofemale lines (IF1, IF2). An exact test for genetic structure (pairwise differentiation test for population differentiation using allelic distribution in the various populations) was used as implemented in GENEPOP [37,38].

### Kin associations (Experiment II)

**Figure 2:**
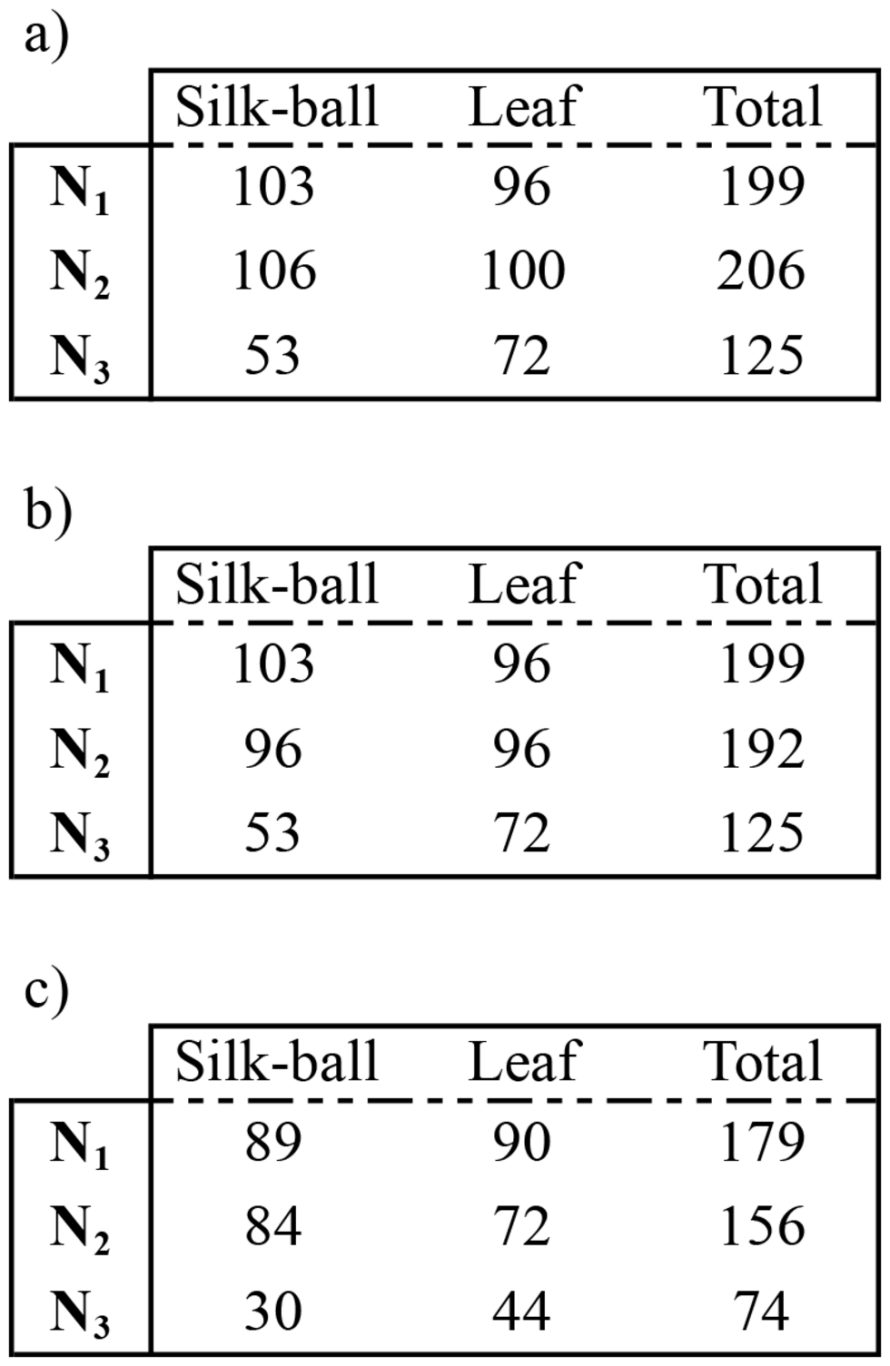
Design of the Experiment *II* using individuals from two isofemale lines (IF1, IF2). Number of females a) Counted in the silk-ball and in the leaf, b) Genotyped and, c) Having a completed genotype is indicated for each repetition (N = 3).

Only individuals sampled in the balls and on the leaves that were fully genotyped (12 loci per replicate) were used in this analysis (N_1_ = 179, N_2_ = 156, N_3_ = 74). Figure 2 illustrates the experimental design. Individuals were classified into three categories according to the origin of their parents: a) both parents came from the isofemale line 1 (IF1), b) both parents came from the isofemale line 2 (IF2), and c) one parent came from line 1 and the other from line 2. This assignment was performed on the basis of individual genotypes at loci having different alleles in the two lines. To determine the loci discriminating the two strains, 30 females sampled from each isofemale line were genotyped. Five loci were found to discriminate among lines, which allowed a straightforward and unambiguous assignment of individuals in the three categories. χ^2^ tests were used to compare the repartition of individuals in the ball and on the leaf per category (and per replicate). 

## Results

### Experiment I - Mite differentiation based on body coloration

#### Distribution and survival of mites in the ball and on the leaf

When including all stages (immatures and adults), both strains (green and red), and all states (dead and alive mites) in the analysis, the number of mites in the silk-ball did not differ (mean ± SE, 279.6 ± 54.9, N = 7) from the number on the bean leaf disc (mean ± SE, 301.6 ± 52.04, N = 7) (paired t-test, t = 0.33, p = 0.75, N = 7). When looking only on mature females (from both strains and all states), the number of mature females was higher on the leaf disc (mean ± SE, 456.1 ± 83.5, N = 7) than in the silk-ball (mean ± SE, 203 ± 51.1, N = 7) (paired t-test, t = 5.27, p = 0.002, N = 7). Males represented only 17.89% ± 2.98 (N = 7) of the population and were homogeneously distributed between the silk-ball and the leaf disc (paired t-test, t = 1.38, p = 0.22, N = 7).

There were as many green as red females in the experimental set-up (bean leaf disc + silk-ball) (paired t-test, t = 2.17, p = 0.07, N = 7). Green and red females were similarly distributed between the silk-ball and the leaf disc (paired t-test comparing the proportion of green females in the silk-ball and in the leaf disc, t = 1.93, p = 0.1, N = 7 and paired t-test comparing the proportion of red females in the silk-ball and in the leaf disc, t = 0.09, p = 0.93, N = 7). The balls were essentially composed of alive mites (84.7 % of alive mites ± 11.4, N = 7). 

### Experiment II - Mite differentiation based on the isofemale line approach

During the Experiment *II*, we collected the balls 4 h after their emergence. There were no dead individuals in the silk-balls. Individuals (males and females) were homogeneously distributed between the silk-ball and the bean leaf disc (paired t-test, t = 0.15, p = 0.89, N = 3). Males accounted for one third (30.33% ± 2.9, N = 3) of the population. 

Among the 20 microsatellites tested, twelve were scored without ambiguity in each replicate (N = 3) and were used in the *F*_*ST*_ analysis. Only individuals fully genotyped at those 12 loci (N_1_ = 179, N_2_ = 156, N_3_ = 74) were kept for the analysis. Pairwise estimates of F_ST_ calculated between pairs of populations were very low for the three repetitions (N_1_ = 0.0255, N_2_ = 0.005, N_3_ = 0.0047). The *F*_*ST*_ value between the two isofemale lines (IF1, IF2) was very high (*F*_*ST*_ = 0.74), indicating that the two isofemale lines constituted two clearly genetically distinct groups. 

The three replicates were constituted of individuals classified in three categories according to the origin of their parents (see Materials and Methods, IF1 × IF1, IF1 × IF2, IF2 × IF2). Individuals per category were homogeneously distributed between the silk-ball and the bean leaf disc for all the replicates (χ^2^ tests, Table 2). Therefore, individuals did not segregate with kin during the formation of the collective ball. 

**Table 2:** Individual distribution per category (according to the origin of their parents: both parents came from the isofemale line 1, IF1 x IF1; one parent came from line 1 and the other from line 2, IF1 x IF2; both parents came from the isofemale line 2, IF2 x IF2) and per repetition (N = 3).

	**N_1_**			**N_2_**			**N_3_**		
**IF**	**Silk-ball**	**Leaf**	**χ^2^**	**Silk-ball**	**Leaf**	**χ^2^**	**Silk-ball**	**Leaf**	**χ^2^**
IF1 x IF1	9	6	0.6	7	6	0.077	3	6	1
IF1 x IF2	33	42	1.08	56	43	1.707	21	27	0.75
IF2 x IF2	47	42	0.281	21	23	0.091	6	11	1.471

Chi-square tests were used to assess whether the repartitions between the ball and the leaf were significantly different per category. IF = isofemale line.

## Discussion

Our study focused on the group dispersal in a subsocial organism, *T. urticae*, and on the conditions leading to such collective behaviour. The influence of the genetic, stage and timing during the formation of collective silk-balls were examined. Two different scenarios were proposed for the silk-ball emergence: mites could disperse with kin (kin selection) or mites could aggregate in the silk-ball with related and unrelated individuals (natural selection). While further field and lab experiments are needed to conclude that kin selection is not involve in silk-ball formation, the results here obtained, suggests that the group size (silk-ball) is a key element to ensure effective dispersal. Instead of leaving individually, mites leave the plant in group (i.e. silk-ball structure) with related and unrelated individuals. Group dispersal may provide advantages with respect to overcoming Allee effect or reducing cost during the dispersal process (e.g. desiccation reduction) [32]. In addition, dispersing with unrelated individuals in the ball group increases genetic with different consequences for the founding population. First, it reduces the risk of inbreeding depression. Indeed, as balls are composed of immature stages, once they reach a new plant they will mature and mate with unrelated individuals. Second, the presence of a diversity of genotypes increases the probability for selecting the adapted to new environmental conditions [39,40]. In many invertebrate species (with extremely variable degree of sociality), individuals form groups and perform collective actions in a variety of contexts such as nest construction and maintenance, colony emigration and defense [41]. 

When overcrowded plants are exhausted, individuals have at least two choices: going in the ball or remaining in the plant. Our study has revealed that half of the population chose the first option and the second half the second one. Two non-exclusive hypotheses can be raised to explain why half of the mites remained on a deteriorated bean plant. First, the behaviour (i.e. forming the ball or remaining on the plant) might result from a different sensitivity or resistance to environmental stress (e.g. food deprivation, desiccation). This could explain why individuals that might be more sensitive to starvation (individuals having different thresholds to leave the plant and go up in the ball) initiate the ball and are trapped in the silk weaved by latter arrivals. Such a behavioural variability can be explained by qualitative and quantitative variations in individual responsiveness [42]. Studies on spider mites support the hypothesis that mites might react different to thresholds of a given stimulus, for example, the attraction to silk [33] or for dispersal [28]. In addition, a genetic component was identified in the case of aerial [28] or ambulatory dispersers [43]. A variety of internal factors such as genetic predisposition, morphology or age can also determine the threshold of each individual. This has been shown in social insects [44-46] but not in spider mites. The second hypothesis is that several balls may be formed successively. Therefore, the mite population would be gradually removed from the infested plant. 

While *T. urticae* individuals can discriminate their kin individually [23-26], they did not segregate with kin at the collective level (silk-ball). At least two distinct hypotheses can be raised to explain these observations. First, the recognition abilities can be overruled by other mechanisms such as aggregation [22,25,47]. Silk as an attractive and cohesion cue [25] could be stronger than discrimination among individuals in a group of mites (as already shown in cockroaches and spiders) [22,47]. Second, behaviour can change when mites are faced with different environmental situations. In conditions encountered during the ball formation (stress, density) the ability of recognition, which modulates the aggregation, seems to be vanished. 

Immature individuals are the most vulnerable stages (e.g. low mobility, high desiccation and predation risk) and are not involved in individual dispersal [48,49]. When food resources are depleted, being in a ball could be the unique option for them to disperse in a protective structure. Different dispersal strategies between adults and juveniles might be due to different responsiveness to starvation. This phenotype-dependent dispersal could have strong implications for population dynamics and for the persistence of populations in fragmented landscapes [50]. Moreover, as *T. urticae* is highly polyphagous (using a wide diversity of host-plants under various climates), the selection of different dispersal strategies between adult and immature individuals can play a role in determining the range-expansion dynamics of this species.

The silk-ball can be compared to a public transport that mites could use to disperse. However, mortality (or the probability of survival) is clearly linked with the time delay between ball formation and dispersal (or harvest in our case). Indeed, four-hours-old balls did only contain living individuals, whereas in one-day-old balls 30% of individuals were dead [32]. Moreover, the percentage of dead individuals within the ball seems to depend on the size of population infesting the plant. In the present experiment, one-day-old balls were composed of a total of 280 mites, much less compared to balls of 790 individuals in [32]. In our previous study, mites had more resources on the bean plant and therefore populations grew bigger [32]. When the population is high, more individuals gather in the ball and increase therefore the number of dead mites. 

Initiating a ball could result from a trade-off between the risk of dying on the plant without any progeny or in the silk-ball, which gives some probability of surviving and laying eggs on a new plant. Under the laboratory conditions used in this study, there are no passing animals (i.e. phoresy) or wind, and the ball is allowed to stay on the wooden stick for 24 or 4 h. While no data are available in the literature about the dispersal capacities of such silk balls in nature, one may assume that the time, distance and direction of collective dispersal depend on various factors such as the position of the ball over the plant, its size but also the features of air currents or the density of phoretic animals. Such dispersal events represent a risk because mites do not control their final destination and several potential unfavourable factors such as inadequate weather, water, predation pressure, and host-plant at the destination might be encountered. It would be interesting to study in the field when, how far, and under which conditions (e.g. wind speed, presence/absence of phoretic animal) the balls go and how much they participate to the dispersal of the mites. Studying the balls’ lifetime seems essential to complete the understanding of this collective behaviour. Measurement of the survival chances of a group of mites transported in such a ball and its success in population growth remains to be explored.

Although our study highlights the importance of the group size in the ball formation, further experiments are needed to fully understand the mechanisms that underlie the emergence and growth of the ball (natural versus kin selection). For example, further laboratory studies on silk-ball formation need to be done using more than one single stick. Indeed, in natural conditions, there would be many stems upon which the mites could climb. It would be interesting to observe whether balls differ when the mites have to choose between different sticks (i.e. ball formation and composition). The presence of several sticks could also demonstrate a change in mites’ behaviour by aggregating themselves with kin on a precise stick. Moreover, field studies could provide precious information on how silk-balls are formed in natural conditions (i.e. population size, genetic composition, ball size and frequencies) and dispersed (i.e. environmental conditions, dispersal distance, survival rate).

## Supporting Information

Supporting Information S1 **Protocol to create isofemale lines of two-spotted spider-mites.**
(DOC)

Supporting Information S2 **Accession numbers and references of the microsatellite loci used to genotype *Tetranychus urticae* mites.**
(DOC)
