# Supplementary material for: Reaching the Ball or Missing the Flight? Collective Dispersal in the Two-Spotted Spider Mite Tetranychus urticae
Source: PLoS One. 2013 Oct 15;8(10):e77573. doi: 10.1371/journal.pone.0077573 (PMC3797074; doi:10.1371/journal.pone.0077573)
Supplement: Supporting Information S1 — Protocol to create isofemale lines of two-spotted spider-mites. (DOC) [file pone.0077573.s001.doc]

Bean leaf discs (14 mm diameter) were placed on wet cotton in Petri dishes (85 mm in diameter, 13 mm deep) with one young adult female (24 h, unmated) by leaf discs (N = 20). Once females had laid a few eggs (at least four eggs) on leaf discs, females were moved to other individual bean leaf discs in a climate room at 10 °C. This low temperature reduced the metabolism of individuals, and the lifetime of females was increased (at least until the maturation of their eggs into adult males). Then, each female was placed with its own sons for mating (climate room at 26 °C, with a relative humidity of 50–60% and a photoperiod of L16:D8). Subsequently, the mated female started to lay eggs and after about 15 days, and the inbred population was created.
