# Supplementary material for: Reaching the Ball or Missing the Flight? Collective Dispersal in the Two-Spotted Spider Mite Tetranychus urticae
Source: PLoS One. 2013 Oct 15;8(10):e77573. doi: 10.1371/journal.pone.0077573 (PMC3797074; doi:10.1371/journal.pone.0077573)
Supplement: Supporting Information S2 — Accession numbers and references of the microsatellite loci used to genotype Tetranychus urticae mites. (DOC) [file pone.0077573.s002.doc]

| **Locus** | **Accession no.** | **Reference** | |
| --- | --- | --- | --- |
| **PCR multiplex set 1 (Annealing temperature: 58°C)** | | |  |
| *TuCA72* | DDBJ-AB263080 | [36] | |
| *TuCT37* | DDBJ-AB263089 | [36] | |
| *Tu11* | AJ419829 | [35] | |
| *Tu27* | AJ419831 | [35] | |
| *TuCA12* | DDBJ-AB263078 | [36] | |
| *TuCT09* | DDBJ-AB263084 | [36] | |
| *TuCA25* | DDBJ-AB263079 | [36] | |
| *TuCT13* | DDBJ-AB263085 | [36] | |
| *TuCT81* | DDBJ-AB263092 | [36] | |
| *TuCT67* | DDBJ-AB263090 | [36] | |
| **PCR multiplex set 2 (Annealing temperature: 52°C)** | | |  |
| *TuCT73* | DDBJ-AB263091 | [36] | |
| *TuCA83* | DDBJ-AB263081 | [36] | |
| *Tu16* | AJ419830 | [35] | |
| *Tu1* | AJ419828 | [35] | |
| *TuCT17* | DDBJ-AB263086 | [36] | |
| *TuCT26* | DDBJ-AB263088 | [36] | |
| *Tu35b* | AJ419832 | [35] | |
| *TuCT18* | DDBJ-AB263087 | [36] | |
| *TuCT04* | DDBJ-AB263083 | [36] | |
| *TuCA96* | DDBJ-AB263082 | [36] | |
